# Ulcerative Esophagitis Caused by Cytomegalovirus During Infectious Mononucleosis in a Young and Immunocompetent Male Patient‐A Case Report

**DOI:** 10.1002/ccr3.70676

**Published:** 2025-07-28

**Authors:** Duje Apostolski, Dietmar Reitgruber, Carina Primus‐Grabscheit, Zsofia Hetzmann, Johann Auer

**Affiliations:** ^1^ Department of Internal Medicine I Hospital St. Josef Braunau Braunau am Inn Austria; ^2^ Department of Internal Medicine I University Hospital Krems Krems an der Donau Austria; ^3^ Department of Pathology Clinic Wels‐Grieskirchen Wels Austria

**Keywords:** cytomegalovirus, esophagitis, immunocompetent patient, infectious mononucleosis

## Abstract

Cytomegalovirus esophagitis mostly occurs in immunodeficient and severely acutely ill patients. Moreover, this diagnosis should be considered in otherwise healthy patients that experience reflux esophagitis symptom worsening under active CMV infectious mononucleosis. Antiviral therapy and screening for immunodeficiency may be considered; however, more studies are needed to support this claim.

**Abbreviations**
CMV: cytomegalovirus
COPD: chronic obstructive pulmonary disease
DNA: deoxyribonucleic acid
EBV: Epstein Barr virus
GI: gastrointestinal
HIV: human immunodeficiency virus
ICU: intensive care unit
IgG, IgM: immunoglobulin G, M
PCR: polymerase chain reaction
PPI: proton pump inhibitor

## 1 Introduction

Cases of cytomegalovirus gastrointestinal disease are mostly apparent in immunocompromised patients [1]. The esophagus is the second most common site after the colon, most frequently presenting as epigastric pain or without symptoms [2]. Until now, there have been only a few cases described in young, immunocompetent patients [3, 4]. In our study, we present the cytomegalovirus esophagitis in a 21‐year‐old, immunocompetent male patient, being the youngest case ever described according to searched literature.

## 2 Case History/Examination

A 21‐year‐old male patient was presented at the hospital St. Josef Braunau in Austria in May 2022 with fatigue, weight loss, and lack of appetite lasting for several weeks. Significant weight loss of more than 10 kg in the last 30 days was reported, as well as the inability to tackle the obstacles of everyday life. The patient reported a brief episode of occasional tobacco use 2 years ago. Family and social history, as well as prior medical history, was without any special features.

In a clinical examination, other than adiposity, no pathological signs could be found. Organomegaly and lymphadenopathy were not palpable.

## 3 Differential Diagnosis, Investigations, and Treatment

Routine laboratory findings showed a normal leukocyte count of 8000/μL with 44% lymphocytosis and 11% atypical lymphocytes proved by manual differentiation. Liver enzymes were three times over the upper reference limit. Other laboratory findings, including inflammation parameters, were in the normal range.

Working diagnosis of viral infection, particularly infectious mononucleosis, was made and therefore a serology of various infectious diseases was undertaken. No signs of active or healed hepatitis B, C, or HIV infection could be detected. Serology of Epstein–Barr virus (EBV), the most common cause of infectious mononucleosis, showed only signs of already healed infection (IgG +, IgM−). A PCR test of the second most common infectious mononucleosis pathogen, cytomegalovirus (CMV), was also performed.

**FIGURE 1:**
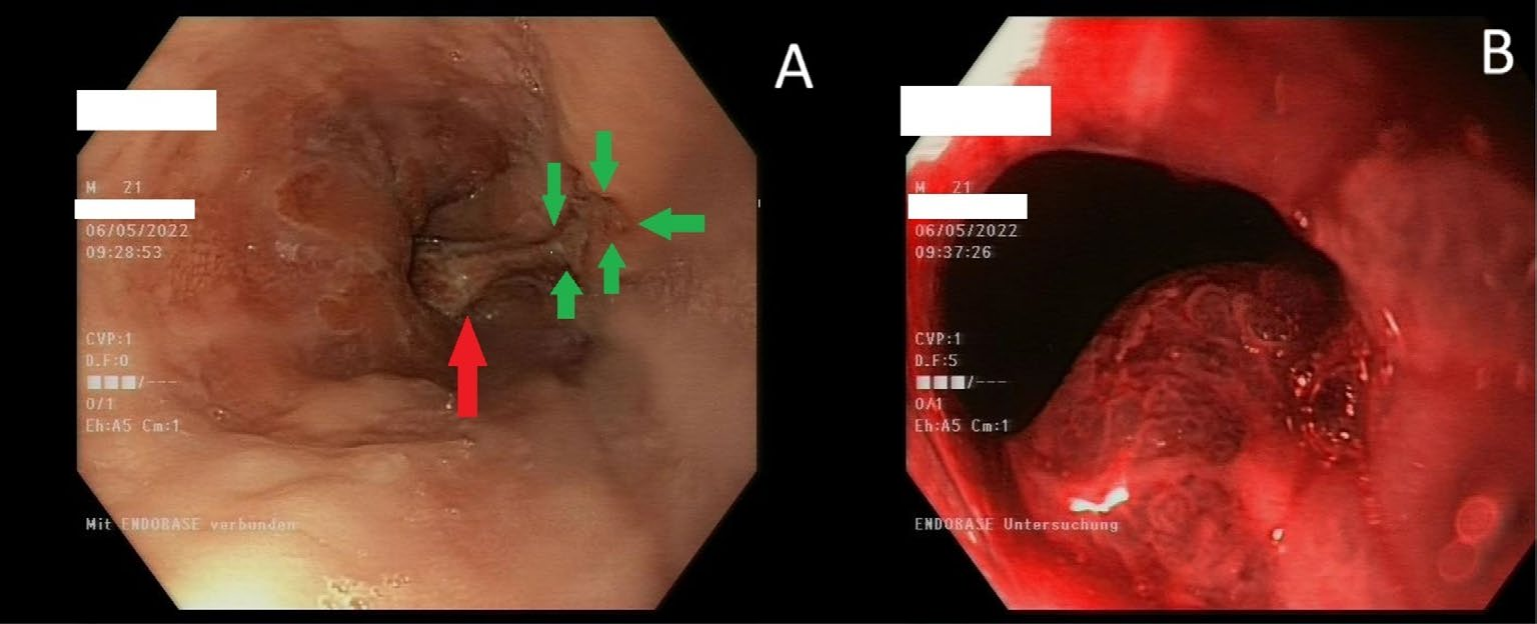
(A) Reflux esophagitis Los Angeles Grade B and C with ulcus (green arrows). Red arrows show beginning of the hyperplastic reactive polyp. (B) Hyperplastic reactive polyp under NBI light.

Finding of splenomegaly (15 × 6 cm) in an abdomen ultrasound was further supporting our suspicion. As the patient was still showing signs of inappetence and epigastric discomfort despite the symptomatic therapy, gastroscopy was performed. A high‐grade reflux esophagitis (Los Angeles Grade B and C), ulcer, and a sessile polypoid structure at the gastroesophageal junction were described (Figure 1). We decided to perform biopsies in the area of the polyp and the ulcer.

As the patient's condition improved during the hospital stay, we discharged him after 4 days with a standard dose of pantoprazole (4 weeks or longer depending on symptoms) and paracetamol. Gastroscopy control in a few months was also suggested if the symptoms do not completely resolve under pantoprazole therapy. Early clinical follow‐up was advised only in the case of worsening symptoms.

On the same day, few hours after the discharge, PCR DNA analysis revealed more than 16,000 DNA copies of cytomegalovirus in peripheral blood, clearly showing active CMV infection, hence confirming our initial workup diagnosis.

**FIGURE 2:**
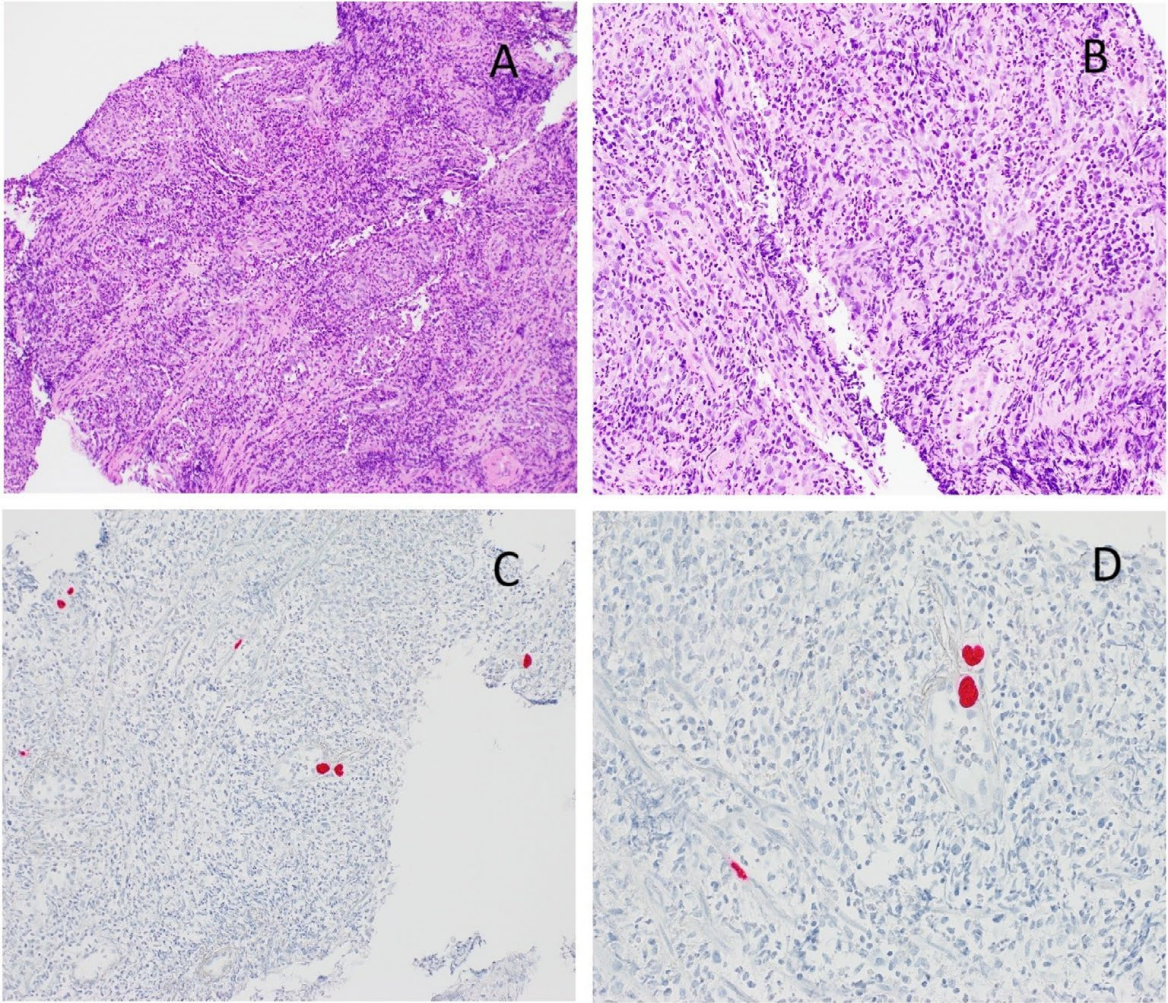
Pathohistological findings. (A and B) Granulation tissue of an ulcer with marked inflammation and capillary vessel proliferation inside of the remaining epithelial tissue 100× and 200× enlargement, respectively. (C and D) Cells inside of an inflammed epithelial tissue at the site of ulcer with positive immunohistochemical reaction against CMV (red dots) presented in 200× and 400× enlargement.

The pathohistological analysis report of the taken biopsies at the site of the ulcus came about 2 weeks after the discharge. It showed a squamous epithelium with inflammatory cells, matching the endoscopical findings of reflux esophagitis. Samples closer to the site of the mentioned polyp and ulcer showed a granulation tissue with strong inflammatory infiltrates and proliferation of capillary vessels. At the time of the probe referral, the pathologist was aware only of the symptoms regarding the gastrointestinal system. However, since the finding of an ulcerative high‐grade esophagitis described in the referral was very unusual for such a young patient, the pathologist decided to expand the routine diagnostic procedure. Therefore, the most common pathogens causing esophagitis were tested. Even though there were no typical core changes in the context of CMV infection, some cells around the ulcer showed a positive immunohistochemical reaction against CMV antibodies (Figure 2). This indicated that the ulcer and surrounding inflammation with the formation of a hyperplastic polyp was at least partially caused by CMV esophagitis.

After revision of the new findings, no therapy was added, as there was no clear data about antiviral therapy of CMV esophagitis in young, immunocompetent patients.

## 4 Outcome and Follow‐Up

In order to obtain a written consent to analyze and publish our findings, we arranged a follow‐up clinical examination about 10 months after the discharge (March, 2023). Except for the fatigue lasting 1 month, the patient reported no other symptoms after the discharge. The proposed pantoprazole therapy was not fully taken by the patient. The patient expressed the wish to repeat a gastroscopy in order to make sure that the findings in the esophagus resolved.

**FIGURE 3:**
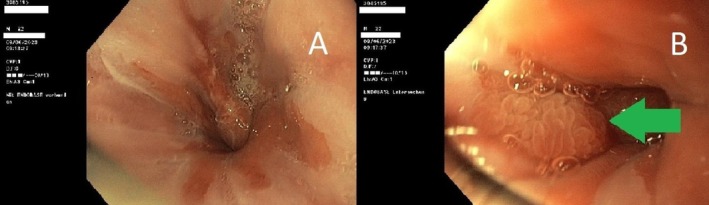
Control gastroscopy 13 months after the discharge. (A) reflux esophagitis Los Angeles Grade A and B. (B) Small mucosal hyperplasia in cardia (arrow) as a part of the reflux esophagitis, with no signs of formerly described polyp.

Therefore, a control gastroscopy and laboratory was scheduled 13 months after the discharge (June, 2023). The findings during gastroscopy showed a marked improvement. A typical mild reflux esophagitis (Los Angeles Grade A and B) as well as a few small mucosal, non‐polypoid elevations at the gastroesophageal junction were described. There were no signs of ulcers and polyps (Figure 3). Routine biopsy samples were taken.

Analyzed tissue of the taken biopsy samples showed only low grade esophagitis and mucosal follicular hyperplasia in cardia, both as part of underlying reflux disease. There were no pathohistological signs of CMV infection.

Control blood count and liver enzymes were in the reference range. No copies of CMV DNA were detected in peripheral blood. Prolonged therapy with a standard dose PPI and preventive measures (weight loss, alcohol abstinence, and diet) were prescribed.

## 5 Discussion

CMV infections of the gastrointestinal tract, especially the esophagus, are rare in immunocompetent young patients. In the case report written by Gravito‐Soares et al. all previously described cases of immunocompetent patients with CMV esophagitis were summarized and reviewed in a table. According to that data, until 2018, only 25 such patients were described, presenting mostly with upper GI bleeding and/or epigastric pain. The real incidence is arguably even lower, considering that almost all listed patients had some serious underlying chronic disease or were acutely seriously ill, usually presenting after emergency surgeries and/or receiving treatment in the intensive care unit, which raises questions about the competence of their immune system at the time of acquiring CMV esophagitis [3].

In the same case report, the youngest ever recorded patient (25 years old) with cytomegalovirus esophagitis was described. Unlike the patients summarized in the mentioned table, this patient didn't have any chronic diseases at the time of CMV esophagitis presentation and was not severely ill. On the other hand, two occurrences of herpes zoster in younger age (7 and 16 years old) according to medical history could arguably show some potential underlying impairments of the immune system [3]. The patient in our case report is obviously younger and on top of that, no childhood diseases were reported in medical history, making the presence of CMV esophagitis even more unlikely.

We decided to prescribe our patient analgetics and PPI without antiviral therapy. There is no published data describing a benefit of the antiviral therapy in infectious mononucleosis in young and immunocompetent patients. To add up, anamnestically, symptoms lasted for a few weeks before the admission, making the antiviral therapy futile from our point of view.

Studies describing antiviral therapy in CMV infections of the GI system always include severely ill patients, mostly needing ICU treatment [4, 5, 6, 7, 8, 9, 10, 11, 12]. The rationale behind therapy lies in severe and even fatal outcomes of CMV esophagitis described in some studies [13, 14]. Nevertheless, such outcomes were not described in immunocompetent patients without underlying acute disease, especially in the younger age. That being said, the therapy is probably required only in severely ill or immunocompromised patients.

**FIGURE 4:**
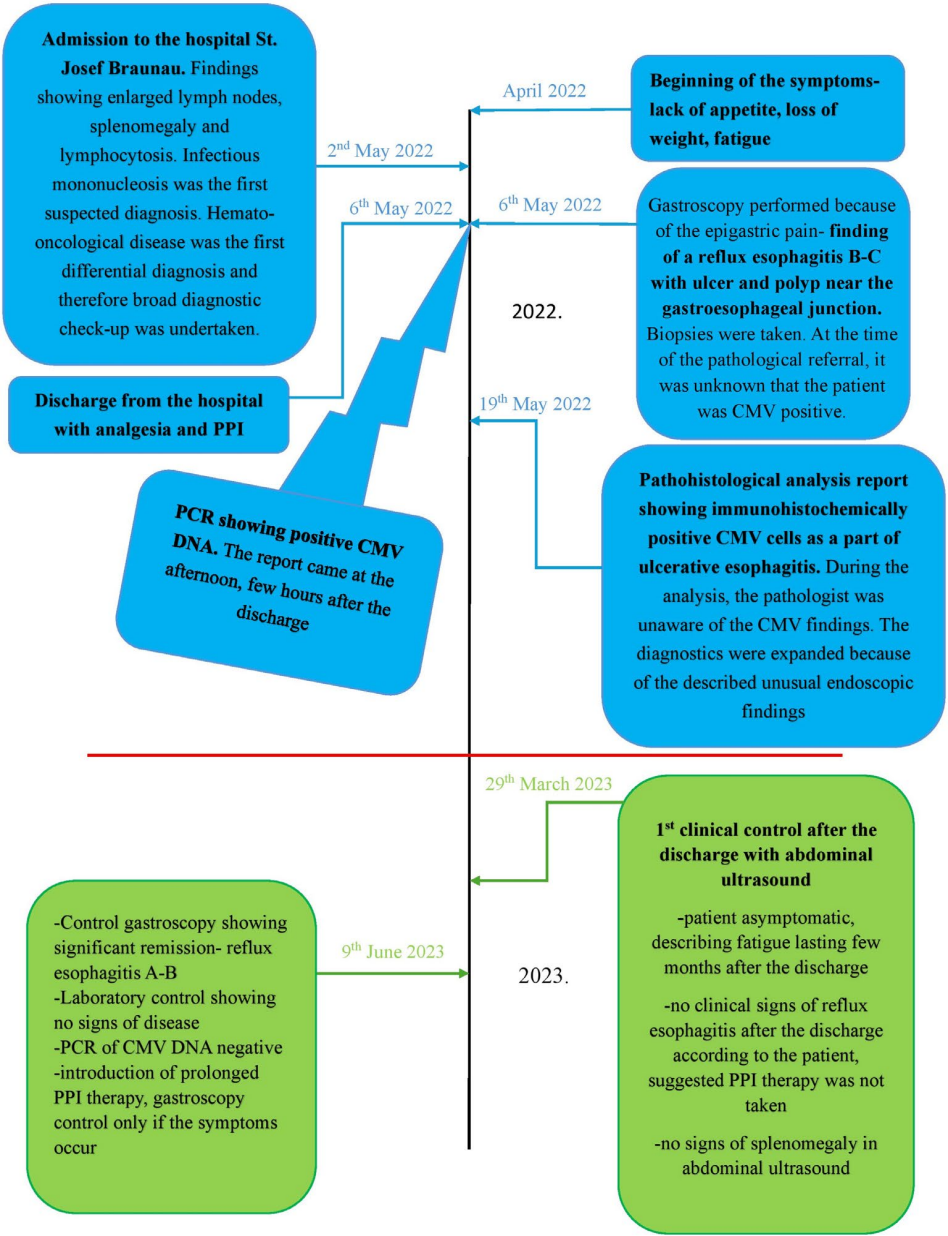
Case report timeline.

Regarding PPI and grade of reflux esophagitis, after revision of findings, PPI therapy was prolonged from 4 to 8 weeks. There are no studies addressing the duration of PPI therapy and frequency of endoscopic controls in this kind of patient, thus we followed the treatment recommendations for the common reflux esophagitis. Unfortunately, as we later found out, the PPI therapy was not enforced by the patient as prescribed. Nevertheless, the control after more than 1 year showed major improvements endoscopically and clinically with complete restitution of laboratory findings (Figure 4).

With the majority of the immunodeficiency causes being excluded, the reason why our patient developed CMV esophagitis remains unknown. We suspect that the patient had a reflux esophagitis before developing infectious mononucleosis, which made him more susceptible to CMV, doing additional damage in the distal esophagus.

Considering the rarity of these cases, the question arises if the patients with diagnosed CMV esophagitis really are immunocompetent and therefore should undergo additional diagnostic procedures and follow‐up to determine possible underlying causes of immunodeficiency. At this moment, there are no studies that could answer this question.

This case report describes an unusual case of the youngest ever immunocompetent patient with diagnosed CMV esophagitis to this date. It might help the clinicians to consider this diagnosis when encountering the similar clinical presentation and medical history. Extended diagnostic procedures to exclude most common immunodeficiency causes can be considered.

## Author Contributions

**Duje Apostolski:** conceptualization, data curation, investigation, methodology, resources, writing – original draft, writing – review and editing. **Dietmar Reitgruber:** conceptualization, supervision, validation, writing – review and editing. **Carina Primus‐Grabscheit:** conceptualization, supervision, validation, writing – review and editing. **Zsofia Hetzmann:** conceptualization, supervision, validation, writing – review and editing. **Johann Auer:** conceptualization, supervision, validation, writing – review and editing.

## Ethics Statement

The patient was well informed about publication and agreed to participate voluntarily, signing the written consent.

## Consent

The patient signed a written consent for publication.

## Conflicts of Interest

The authors declare no conflicts of interest.

## Data Availability

All data is available at any time when requested.
